# Effect of Energy-Dependent Proton Irradiation in Thin-Film YBa_2_Cu_3_O_7−δ_ Superconductor

**DOI:** 10.3390/ma18214845

**Published:** 2025-10-23

**Authors:** Trevor Harrison, Joshua Kim, Katharina Cook, Hope Weeda, Joseph Fogt, Nolan Miles, Kyuil Cho

**Affiliations:** Department of Physics, Hope College, Holland, MI 49423, USA; trevor.harrison@hope.edu (T.H.); joshua.kim@hope.edu (J.K.); kc7489@hope.edu (K.C.); hope.weeda@hope.edu (H.W.); joseph.fogt@hope.edu (J.F.); nolan.miles@hope.edu (N.M.)

**Keywords:** high-temperature superconductor, cuprates, YBCO, thin-film, proton irradiation, disorders, resistivity, Abrikosov-Gor’kov theory, d-wave

## Abstract

The superconducting properties of YBa_2_Cu_3_$O_{7-\delta }$ thin films were investigated by conducting 1.7 MeV proton irradiations with a total fluence of $2.64\times 10^{17}$ ${p/\mathrm{cm}}^{2}$. The superconducting critical temperature ($T_{c}$) was reduced from 89.4 K to 10.1 K. The experimental procedure was similar to a previous study (0.6 MeV proton irradiation). We compared the effectiveness of $T_{c}$ suppression by varying the proton energy from 0.6 to 1.7 MeV and found that in general both protons of 1.7 MeV and 0.6 MeV were effective in suppressing the $T_{c}$ of YBCO. In particular, both results were consistent with the theoretical expectation (generalized d-wave AG theory) when a zero-temperature London penetration depth ($\lambda_{0}$) = 215 nm is assumed for thin-film YBCO. For heavily irradiated cases (more than 80% $T_{c}$ suppression), however, 1.7 MeV protons were more effective in suppressing $T_{c}$ than 0.6 MeV protons. This can be understood by the fact that in the thin-film limit, higher-energy protons tend to produce less clustered point defects while lower-energy protons tend to create agglomeration of point defects.

## 1. Introduction

The discovery of superconductivity in cuprate compounds in 1986 opened a new era of high-temperature superconductivity [1,2]. Due to their exceptional properties (high-$T_{c}$, $J_{c}$, and $H_{c2}$), they became important materials for novel technologies [3,4]. Among them, YBa_2_Cu_3_$O_{7-\delta }$ (YBCO) is one of the most studied compounds. While its Cooper pairing mechanism still remains under debate, it is widely accepted that the optimally doped YBCO has $d_{x^{2}-y^{2}}$ wave pairing symmetry where the energy gap vanishes in nodal directions [5].

There are many different ways to investigate the pairing symmetry of a superconductor. One of the effective methods is to introduce artificial disorders into the crystal structure of the superconductor and study the response of its superconducting properties. According to Anderson’s theorem, non-magnetic disorders are not effective in suppressing the superconductivity of isotropic s-wave superconductors [6]. However, magnetic disorders are effective in suppressing the superconductivity of s-wave superconductors (the so-called Abrikosov–Gor’kov theory, or AG theory) [7,8]. In an anisotropic d-wave superconductor such as YBCO, non-magnetic disorders are also effective in suppressing the superconductivity [9]. Openov et al. generalized the AG theory for the case of a symmetry more complex than s-wave of the order parameter and explained the effect of non-magnetic disorders on the property of d-wave superconductors [10].

High-energy particle irradiation has been used as a useful tool to introduce controlled defects into various types of superconductors (bulk single crystals, thin-films, tapes, etc.). It has been used to enhance the critical current ($J_{c}$) of a superconducting material [11] and to test superconducting microwave filter in space-like environments [12]. Another important use is to study the order parameters of various superconductors [13,14,15]. Experimental confirmations of the generalized d-wave AG theory have also been conducted through high-energy particle irradiation. Different types of particles generate different forms of defects [16]. Electron irradiation has been known to be most effective in the generation of point-like atomic-scale defects due to its low rest mass, resulting in a rapid suppression of superconductivity [17,18,19,20]. Other particles, such as protons and heavy ions, have previously been shown to be effective in creating cascading or columnar defects which have been known to be significantly less effective in suppressing superconductivity [21,22,23,24]. However, neither of these results (electron or others) successfully reproduced the theoretical expectations (generalized d-wave AG theory). Some results have reproduced similar behavior, but rely on a more qualitative analysis rather than direct quantitative agreement. These results were explained in terms of the different quality of each sample, different plasma frequencies [9,25], different ratios between in-plane and out-of-plane defects [26], and different electron correlations [27,28,29]. In our previous study, we conducted 0.6 MeV proton irradiation of a thin-film YBCO superconductor using Hope College’s particle accelerator (1.7 MV tandem Van de Graaff electrostatic accelerator) [30]. This experiment produced results that were quantitatively closer to the theoretical expectation than any previous results. Therefore, we concluded that as the sample thickness approached the thin-film limit, the cascading defects produced by proton irradiation would become negligible, and the atomic point-like defects would dominate.

In this article, we conducted higher-energy (1.7 MeV) proton irradiation in a YBCO thin-film superconductor and compared the result with the previous 0.6 MeV experiment. We found that in general, both 1.7 MeV and 0.6 MeV protons were equally effective in suppressing the superconductivity except for the most heavily irradiated cases (more than 80% $T_{c}$ suppression). For the most heavily irradiated cases, the 1.7 MeV proton was more effective in suppressing $T_{c}$ than the 0.6 MeV proton, indicating that the 0.6 MeV proton is more prone to create agglomeration of point defects than the 1.7 MeV proton. Furthermore, we found that both results are consistent with the theoretical expectation when the larger London penetration depth value ($\lambda_{0}$ = 215 nm) was assumed for thin-film YBCO superconductors.

## 2. Material and Methods

### 2.1. YBCO Thin Film and Resistance Measurements

The YBCO thin film (≈567 nm thick) was epitaxially grown on a lanthanum aluminate (LaAlO_3_, or LAO) substrate. The sample was originally fabricated as resonators in commercial microwave filters for wireless base stations [31]. This thin-film sample shows $T_{c}$≈ 89.4 K, indicating that its superconducting property is close to the bulk single-crystalline sample of $T_{c}$≈ 93 K [2]. This sample is of the same batch as a previous study with 0.6 MeV proton irradiation [30].

**Figure 1:**
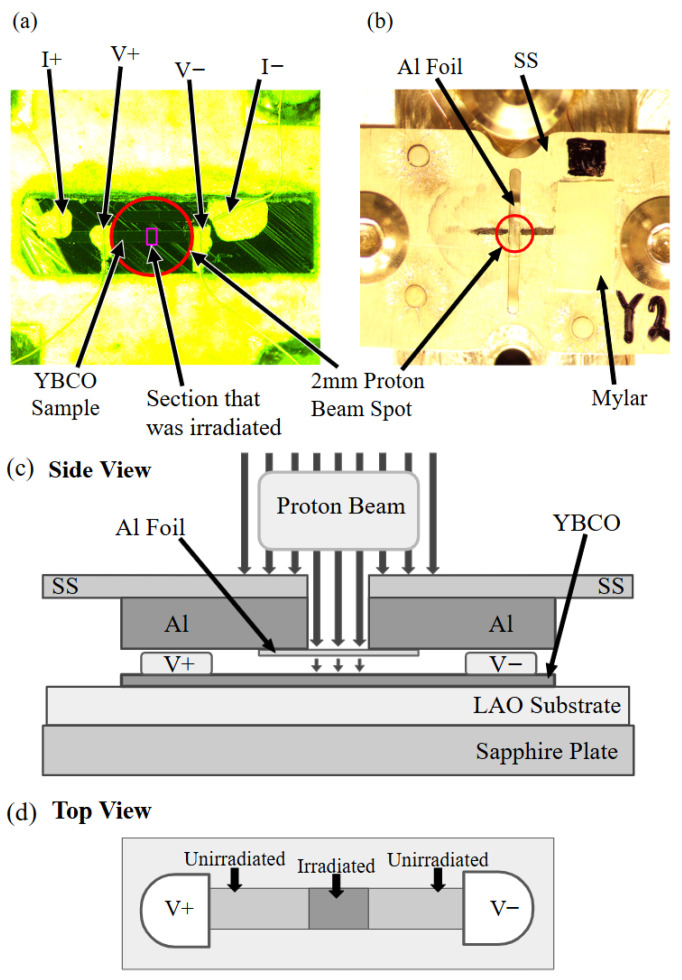
YBCO sample mounted in the sample holder. (**a**) The rectangle on the diagram denotes the section of YBCO that was irradiated. The circle on the diagram indicates the 2 mm diameter beam spot on the sample. (**b**) A stainless steel (SS) beam shield was placed above the sample, reducing the beam width to exactly 0.5 mm. A Mylar scintillator is placed on the beam shield for calibration of the homogeneous 2 mm diameter proton beam. A 50 μm Al energy degrader is placed to reduce the beam energy from 2.85 MeV to 1.7 MeV. (**c**) Side view of the sample holder including the SS beam shield above the YBCO sample, which is grown on the LAO substrate mounted on a sapphire plate. (**d**) Top view of YBCO sample showing the portions of the unirradiated and irradiated parts of the sample. Proton irradiation is homogeneous in the irradiated section of the sample.

The in-plane resistance of the YBCO thin film was measured using a standard four-probe technique as shown in Figure 1. The dimensions of the measured part of the sample are 1.835 (±0.014) mm × 0.263 (±0.001) mm × 567 (±2) nm. Four electrical contacts made of fine gold wires were adhered to the thin film using silver paste.

### 2.2. Energy Degrader and Homogeneous Proton Beam

**Figure 2:**
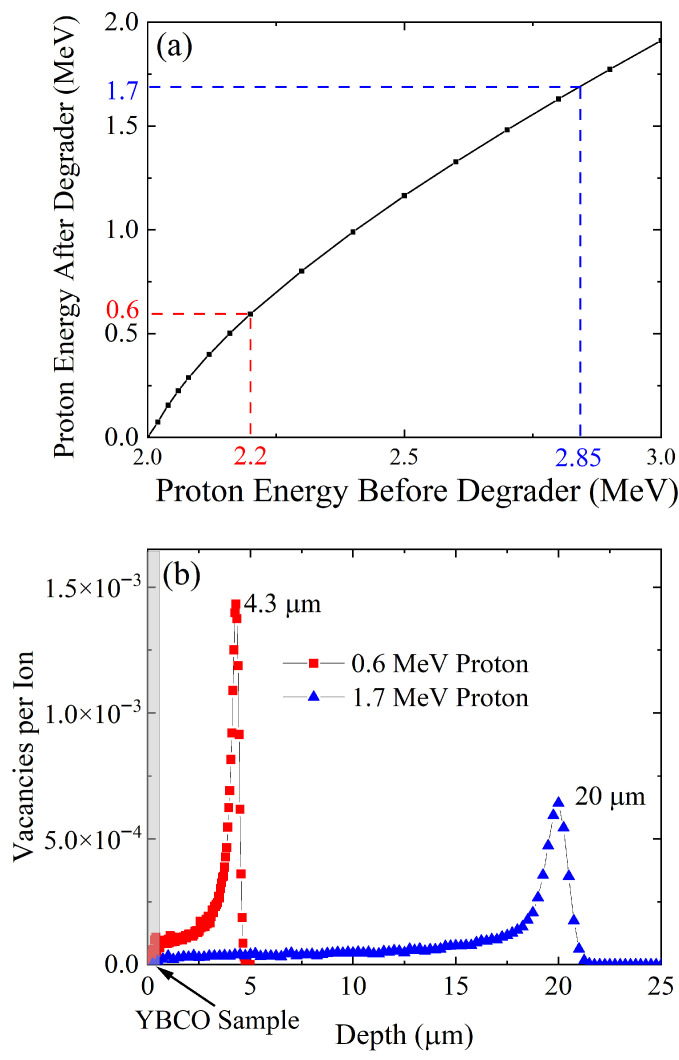
(**a**) The proton beam energy before and after the 50 μm Al energy degrader. The blue and red dotted lines denote the beam energies of the current (1.7 MeV) and previous experiments (0.6 MeV), respectively. These data are calculated using SRIM simulation [32]. (**b**) TRIM simulation results that show vacancies-per-ion along depth for 1.7 and 0.6 MeV protons. Implantation depths for 1.7 MeV and 0.6 MeV protons are about 20 μm and 4.3 μm, respectively. Since the thickness of the YBCO thin film is 567 nm, the protons will be implanted into the LAO substrate. From the simulation, it is clear that a 0.6 MeV proton is about three times more effective in creating defects in YBCO thin film than a 1.7 MeV proton.

The proton beam was produced by Hope College’s particle accelerator (1.7 MV tandem Van de Graaff electrostatic accelerator) which is capable of generating proton beams of up to 3.4 MeV kinetic energy. The irradiation was performed at $6\times 10^{-8}$ torr at room temperature. This irradiation was conducted along the c-axis of the YBCO sample. A 1.7 MeV proton beam was generated by using a 50 μm thick aluminum energy degrader. Using SRIM calculation as shown in Figure 2, we confirmed that if a 2.85 MeV beam passes through a degrader of this thickness, the beam on our target would be 1.7 MeV. This degrader was held in place under the stainless steel (SS) irradiation shield which shielded the entire sample from irradiation besides a 0.5 mm slit on the target area as seen in Figure 1a–c. We tuned the beam to a 2 mm diameter beam spot using the Mylar strip atop the sample mount. We checked that this beam was homogeneous by capturing images of the beam spot and performing an intensity analysis. We tuned the beam until the intensity of the scintillator image was approximately 2 mm across with an even peak. We calibrated the image using the 2 mm by 2 mm square next to the Mylar scintillator as seen in Figure 1b. This careful calibration was essential to ensuring a homogeneous proton beam. The irradiation was divided into 30 min increments, during which we measured the beam current and checked the homogeneity of the beam. Using these current calculations, we estimated the fluences on target as shown in the results. The current on target was kept to about 10 nA to avoid sample heating. During irradiation, we experienced some charge buildup issue in the sample. This resulted in fractures occurring along the silver paste line in the YBCO, which destroys the sample. We avoided this effect by grounding four electrical contacts to the metallic sample holder during the irradiation, which eliminated the rapid charge buildup and discharging issue.

## 3. Results and Discussion

**Figure 3:**
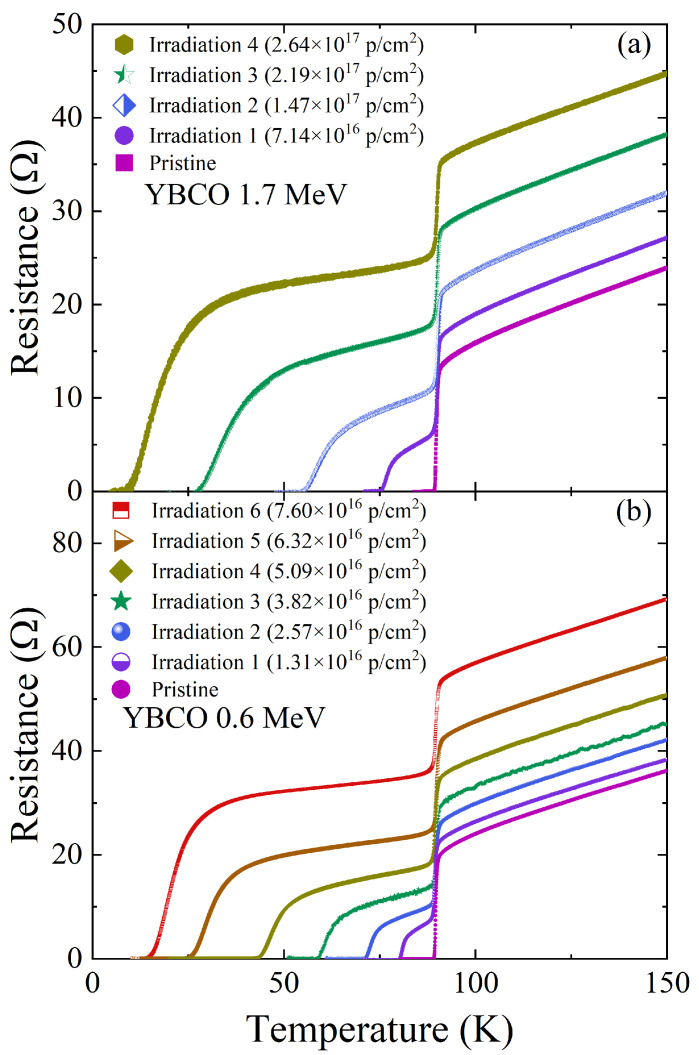
Temperature-dependent resistance data upon a series of (**a**) 1.7 MeV and (**b**) 0.6 MeV proton irradiations. The fluences shown in legends are initially measured by a Faraday cup and corrected using the Rutherford Back-Scattering calibration procedure [33]. For both cases, we see a linear increase in the normal-state resistance upon proton irradiations which is consistent with Matthiessen’s rule.

**Figure 4:**
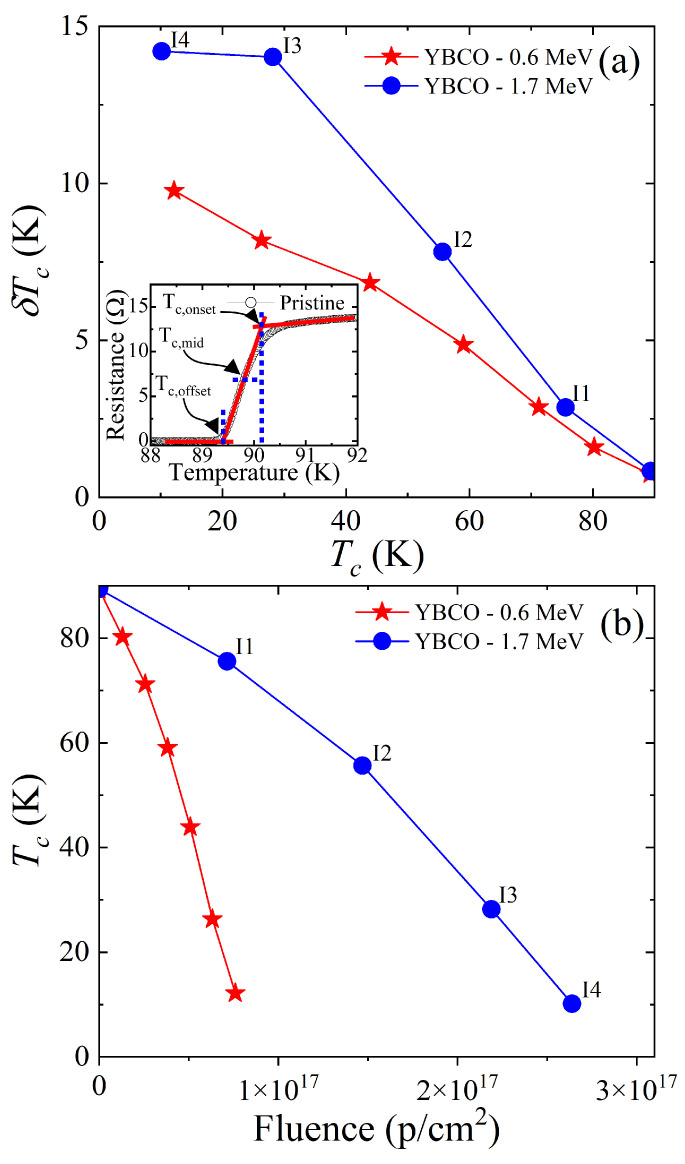
(**a**) Broadening of the superconducting transition ($\delta T_{c}=T_{c,onset}-T_{c,offset}$) for 1.7 MeV and 0.6 MeV proton irradiations. $T_{c,onset}$ and $T_{c,offset}$ are defined in the inset. The horizontal axis is the $T_{c,offset}$, and the vertical axis is the irradiation’s associated broadening. We see much greater broadening in the 1.7 MeV irradiation. (**b**) $T_{c,offset}$ measured for various fluences on 0.6 MeV irradiated and 1.7 MeV irradiated samples. The 1.7 MeV proton beam requires about three times more fluence in order to suppress the superconductivity compared to the 0.6 MeV proton beam. This is consistent with TRIM simulation shown in Figure 2.

Five resistance measurements and four 1.7 MeV proton irradiations were alternatively conducted in the same YBCO thin film. The measured resistance is plotted in Figure 3a in comparison to the previous study of 0.6 MeV proton irradiations (Figure 3b). All of these data show two superconducting transitions except for the pristine cases. These double transitions occur since only part of the area in which the resistance was measured was irradiated as described in Figure 1d. The $T_{c}$ of the unirradiated part does not change while the $T_{c}$ of the irradiated part decreases with increasing fluence. The normal state resistance above $T_{c}$ linearly increases upon irradiation following Matthiessen’s rule. This increase indicates that the number of defects in the irradiated part of the sample increases gradually upon irradiation. The rate of $T_{c}$ suppression with respect to the fluence is about three times larger for 0.6 MeV proton irradiation than for 1.7 MeV proton irradiation (Figure 4b). This is because as we increase the energy of the proton beam, we simultaneously increase the implantation depth. The greater implantation depth means that less energy per proton is deposited into our thin-film sample. As shown in Figure 2b, it is evident that the number of defects created in the thin-film YBCO sample is about three times smaller for 1.7 MeV protons than for 0.6 MeV protons. This is consistent with the fact that 1.7 MeV proton irradiation needs about three times more fluence than 0.6 MeV proton irradiation to achieve the similar $T_{c}$ suppression as shown in Figure 4b. Figure 4a shows the broadening of superconducting transition upon irradiation. This broadening is caused by the spacing between the aluminum energy degrader and the thin-film surface being about 0.7 mm (1.7 MeV proton) and 0.5 mm (0.6 MeV proton). In the future, we plan to redesign the sample holder to decrease this spacing to 0.2 mm in order to minimize the broadening effect.

The increase in normal-state resistance is caused by the resistance increase in the irradiated part of the sample. Thus, we can convert the resistance increase to the resistivity increase by considering the volume of the irradiated part of the sample. Because the normal state resistance increases linearly across all temperature regions above the superconducting transition, the resistance value at 125 K was chosen to calculate the resistivity increase ($\Delta \rho_{125K}$).

**Figure 5:**
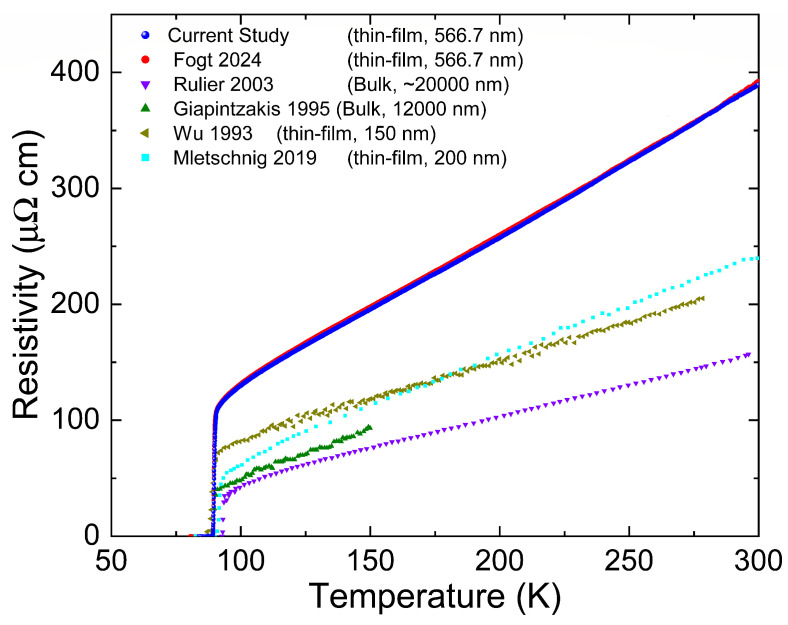
The pristine resistivity of the 0.6 MeV and 1.7 MeV samples. There is little to no difference in the sample resistivity, indicating the samples are nearly identical. Normal-state resistivity in the current YBCO study is higher than previous studies [16,17,18,21,30].

**Figure 6:**
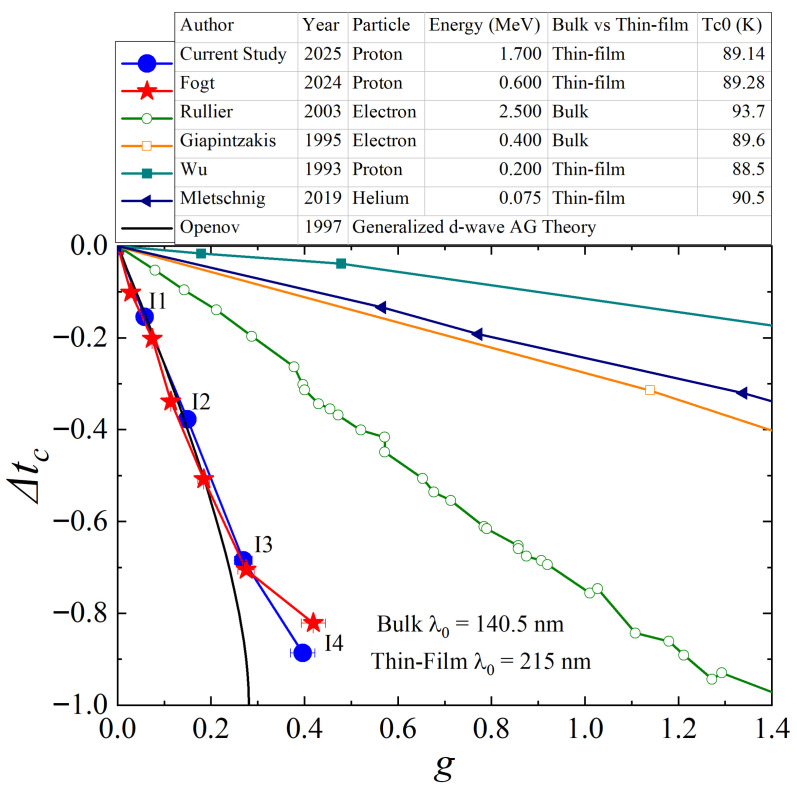
$T_{c}$ ($\Delta t_{c}=\left(T_{c}-T_{c0}\right)/T_{c0}$) as a function of *g* (dimensionless scattering rate) for previous studies and current 1.7 MeV proton irradiation study [16,17,18,21,30] and theoretical expectations [25].

**Figure 7:**
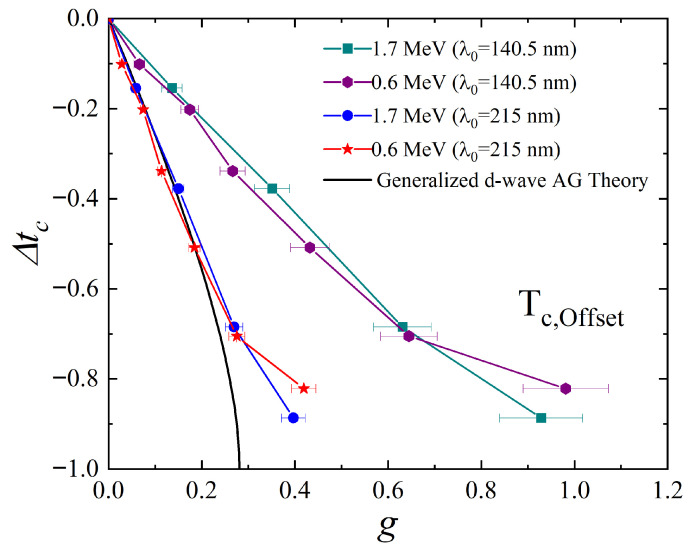
Normalized $T_{c}$ ($\Delta t_{c}=\left(T_{c}-T_{c0}\right)/T_{c0}$) as a function of *g* (dimensionless scattering rate) upon 1.7 MeV and 0.6 MeV proton irradiation. Since *g* depends on the London penetration depth ($\lambda_{0}$), we used two values commonly used for bulk single-crystalline (140.5 nm) and thin-film YBCO (215 nm). When $\lambda_{0}$ = 215 nm is used, both 1.7 and 0.6 MeV proton irradiation results closely follow the theoretical expectation. For the heavily irradiated cases (more than 60 % $T_{c}$ suppression), both results start deviating from the theoretical expectation. Particularly for the most heavily irradiated cases (more than 80% $T_{c}$ suppression), there is a clear difference between 1.7 MeV and 0.6 MeV irradiation.

According to Openov [25], the generalized AG theory for the case of the non-magnetic disorders in d-wave superconductors can be written as follows,

(1) $$-ln\left(t_{c}\right)=\Psi \left(\frac{1}{2}+\frac{g}{2\;t_{c}}\right)-\Psi \left(\frac{1}{2}\right),$$

where $t_{c}$ is $T_{c}$/$T_{c0}$, $T_{c0}$ is the initial $T_{c}$ before the disorders are added, and *g* is the dimensionless scattering rate. $t_{c}$ asymptotically goes to zero as *g* approaches 0.28. Using the Drude model [13,34], *g* can also be written in terms of the residual resistivity ($\rho_{0}$) as follows,

(2) $$g=\frac{\hbar \;\rho_{0}}{2\pi k_{B}\mu_{0}T_{c0}\lambda_{0}^{2}},\;$$

where $\rho_{0}$ is the residual resistivity at *T* = 0 K of the irradiated part of the YBCO sample, $T_{c0}$ is the critical temperature of the pristine sample, and $\lambda_{0}$ is the zero-temperature London penetration depth of the pristine sample. Due to the high $T_{c}$ of the YBCO sample, it is difficult to estimate the exact residual resistivity. If the normal-state residual resistivity is very small, $\rho_{0}$ in Equation (2) can be replaced by Δρ. Indeed, the linear approximation of normal-state resistivity in our previous study [30] suggests very small residual resistivity at *T* = 0 K (≈8 μΩcm) and the current YBCO sample is almost identical to the previous sample as shown in Figure 5. By replacing $\rho_{0}$ in Equation (2) with Δρ, *g* can be rewritten as follows:

(3) $$g\approx \frac{\hbar \;\Delta \rho_{125K}}{2\pi k_{B}\mu_{0}T_{c0}\lambda_{0}^{2}},\;$$

where $\Delta \rho_{125K}$ is the resistivity increase at *T* = 125 K of the irradiated part of the YBCO sample. This calculation requires a value for the London penetration depth, which we are not able to measure in our lab. Therefore, we surveyed experimental values of $\lambda_{0}$ from the previous studies, such as 1460±150 Å [35], 1550 Å [36], 1990±200 Å [37], and 2150 Å [38]. Among them, we used 2150 Å [38] for thin-film single-crystalline YBCO samples and 1405±92 Å [39] for bulk single-crystalline YBCO samples in Figure 6. The YBCO single-crystallined thin films on LAO substrate are structurally less perfect than YBCO bulk single crystals due to strains on the surface. Therefore, strong scattering of the charge carriers, characterized by the mean-free path (*l*), is expected to extend the penetration depth in thin-film samples similar to that predicted by Tinkham [40] for “dirty” superconductors. Thus, a large $\lambda_{0}$ of 215 nm is reasonable for thin-film samples. For the purpose of comparison, we used both London penetration depth values of 140.5 nm and 215 nm to plot our results in Figure 7. When $\lambda_{0}$ = 215 nm is used, both 1.7 MeV and 0.6 MeV results closely follow the theoretical expectation. However, the heavily irradiated results (more than 80% $T_{c}$ suppression) start deviating from the theoretical expectation. This indicates a development of extended defects where the nearby point defects agglomerate at high fluence as mentioned by Wu et al. [21]. For the most heavily irradiated cases, furthermore, $T_{c}$ suppression upon 0.6 MeV proton irradiation is not as effective as 1.7 MeV proton irradiation. This suggests that lower-energy protons are more prone to creating a cluster of point defects even in the thin-film limit than the higher-energy protons.

Figure 6 compares the relation between $T_{c}$ and *g* of the current study with previous irradiation studies [16,17,18,21,30] and theoretical expectations [25]. Note that different penetration depth values are used for bulk single crystals and single-crystalline thin films. The current proton irradiation study is the one that most agrees with the theoretical expectation. In particular, it is surprising that the current proton irradiation is more effective in suppressing superconductivity than the electron irradiation. It can be partially understood that the dominant form of defects caused by proton irradiation shifts from cascade defects to atomic-size point defects as the implantation depth of the proton becomes much longer relative to the sample thickness. However, we currently cannot explain why our results are better than the electron irradiation results. One possible hint can come from a previous YBCO study conducted by Cho et al. [20]. In that article, they found that the higher-energy electron (>1 MeV) has finite scattering cross sections for all atomic sites while the lower-energy electron (<1 MeV) has zero scattering cross sections for a heavy element (Ba). Since $T_{c}$ suppression rate is higher for higher-energy electron beams, they suggested that it is important to have defects in all atomic sites to effectively suppress $T_{c}$. In this regard, the proton is much better than the electron. This should be further investigated. In addition, the current study is indirect evidence of defect dynamics in thin-film YBCO. This can also be further investigated using more direct probes such as TEM and XRD.

## 4. Conclusions

We conducted 1.7 MeV proton irradiation in a YBCO thin film and compared the result with our previous study (0.6 MeV proton irradiation). We found that both 1.7 MeV and 0.6 MeV irradiations were effective in suppressing superconductivity. For the most heavily irradiated cases (more than 80% $T_{c}$ suppression), 0.6 MeV irradiation was less effective in suppressing the superconductivity than 1.7 MeV. This indicates that 0.6 MeV irradiation produced more agglomeration of point defects for the heavily irradiated cases. Furthermore, when $\lambda_{0}$ = 215 nm was assumed, both 1.7 MeV and 0.6 MeV results were consistent with the theoretical expectation except for the most heavily irradiated cases.

## Data Availability

The original contributions presented in this study are included in the article. Further inquiries can be directed to the corresponding author.
